# Effects of Blackcurrant Extract and Partially Hydrolyzed Guar Gum Intake on Gut Dysbiosis in Male University Rugby Players

**DOI:** 10.3390/microorganisms13071561

**Published:** 2025-07-02

**Authors:** Hiroto Miura, Machi Oda, Kanako Abe, Hiromi Ikeda, Mami Fujibayashi, Naoko Oda, Tomohiro Segawa, Aya Abe, Natsumi Ueta, Takamitsu Tsukahara, Tomohisa Takagi, Yuji Naito, Ryo Inoue

**Affiliations:** 1 Laboratory of Animal Science, Department of Applied Biological Sciences, Faculty of Agriculture, Setsunan University, Osaka 573-0101, Japan; hiroto.miura@setsunan.ac.jp (H.M.); machi.oda@edu.setsunan.ac.jp (M.O.); kanako.abe@jm.setsunan.ac.jp (K.A.); h-ikeda@azabu-u.ac.jp (H.I.); 2 Department of Food Science and Human Nutrition, Faculty of Agriculture, Setsunan University, Osaka 573-0101, Japan; mami.fujibayashi@setsunan.ac.jp (M.F.); naoko.oda@setsunan.ac.jp (N.O.); 3 Division of Physical and Health Education, Setsunan University, Osaka 572-8508, Japan; tomohiro.segawa@setsunan.ac.jp; 4 Taiyo Kagaku Co., Ltd., Yokkaichi 510-0844, Japan; aabe@taiyokagaku.co.jp; 5 Morishita Jintan Co., Ltd., Osaka 540-8566, Japan; n-ueta@jintan.co.jp; 6 Kyoto Institute of Nutrition & Pathology, Kyoto 610-0231, Japan; tsukahara@kyoto-inp.co.jp; 7 Department of Molecular Gastroenterology and Hepatology, Graduate School of Medical Science, Kyoto Prefectural University of Medicine, Kyoto 602-8566, Japan; takatomo@koto.kpu-m.ac.jp; 8 Department of Human Immunology and Nutrition Science, Kyoto Prefectural University of Medicine, Kyoto 602-8566, Japan; ynaito@koto.kpu-m.ac.jp

**Keywords:** gut microbiota, blackcurrant, partially hydrolyzed guar gum, rugby, dysbiosis

## Abstract

Our previous study reported that male university rugby players tended to have a gut with a dysbiotic environment, characterized by abundant pathobiont bacteria and an accumulation of succinate, when compared with age-matched, non-rugby playing healthy males. In the present study, we conducted a randomized, double-blinded, placebo-controlled experiment to evaluate the potential of blackcurrant extract and/or partially hydrolyzed guar gum (PHGG) to improve the gut environment of university rugby players. Participants were supplemented with blackcurrant extract and/or PHGG or a placebo for 4 weeks. Beneficial gut bacteria such as *Megasphaera* spp. tended to increase (*p* < 0.10) and *Bifidobacterium* spp. increased (*p* < 0.05) with the intake of blackcurrant extract and/or PHGG. A subgroup analysis further indicated that, unlike in those with a eubiotic gut environment, the dietary supplements also increased the number of beneficial gut bacteria such as *Phascolarctobacterium* spp. (*p* < 0.10) and *Faecalibacterium* spp. (*p* < 0.10) and fecal SCFA concentrations (*p* < 0.05) in participants with a possible dysbiotic gut environment. However, a synergistic effect between blackcurrant extract and PHGG was not clearly observed. Although further investigation is recommended, it was concluded that blackcurrant extract and PHGG can at least be used as functional materials to improve gut dysbiosis in university rugby players.

## 1. Introduction

Humans harbor dense and diverse microbial populations in their hindgut, and these gut microbes and their metabolites have been demonstrated to affect the various physiological functions of the host [1]. Therefore, the state of the gut microbiota and their metabolites seems to be associated with host health, including an abnormal state, known as dysbiosis, potentially leading to various gastrointestinal and systemic diseases [1]. Short-chain fatty acids (SCFAs) are well known to be key bacterial metabolites that contribute to regulating the absorption of water, minerals, and nutrients, inducing immune modulation, and mediating inflammation in the host hindgut [2,3,4]. Therefore, the concentrations of fecal SCFA are frequently regarded as indicators of gut health status. Indeed, a growing number of studies have reported that patients with gastrointestinal diseases, such as inflammatory bowel disease, show disturbances of the gut microbial population and significantly lower concentrations of fecal SCFA when compared with healthy controls [5,6,7,8].

Our previous study reported that male university rugby players tended to have a gut with a dysbiotic environment characterized by abundant pathobiont bacteria and an accumulation of succinate compared with age-matched, non-rugby-playing healthy males [9]. Although it was beyond the scope of the present study, a dysbiotic gut could have been attributed to an unbalanced diet and high-intensity exercise. Generally, to meet an optimal energy level and restore muscle and liver glycogen, athletes tend to consume large amounts of carbohydrates and protein while keeping the ingestion of dietary fiber low to prevent gastrointestinal disturbances such as gas and bloating [10,11]. However, such a dietary pattern is reported to reduce gut microbial diversity and functionality, leading to gut dysbiosis [10,11]. Exercise is also a factor affecting the gut environment. Although simple regular exercise has a positive effect on gut health [12,13], high-intensity exercise is reported to have an adverse effect on it. Specifically, high-intensity exercise can induce increased intestinal permeability and local inflammation resulting from ischemia–reperfusion, altered gut motility and transit, and site-specific oxidative stress, leading to bacterial translocation and an overgrowth of pathobiont bacteria [14,15,16].

In the present study, we focused on blackcurrant extract and partially hydrolyzed guar gum (PHGG) as candidates for functional materials to improve the gut environment of university rugby players. Blackcurrant contains abundant anthocyanins that are bioactive compounds with antioxidant and anti-inflammatory properties [17]. Studies have reported that blackcurrant anthocyanins exert beneficial effect on the host health via improvements to the gut microbial composition and SCFA production [18,19]. PHGG is a prebiotic dietary fiber obtained from the endosperm of the guar bean. Studies have reported that PHGG intake helps to increase the amount of beneficial butyrate-producing bacteria and promote SCFA production in animal and human guts [20,21,22]. Therefore, in the present work, we conducted a randomized, double-blinded, placebo-controlled experiment to evaluate the potential of blackcurrant extract and PHGG to improve the gut health of athletes.

## 2. Materials and Methods

### 2.1. Ethics Statements and Study Participants

The present study was registered in the UMIN Clinical Trial Registry (UMIN000044527) and approved by the ethical committee of Setsunan University, and conducted as per their guidelines (Approval Number: 2021-005, approval dates: 8 May 2021). Written informed consent was obtained from all participants. The enrollment of subjects and sample collection were conducted from June 2021 to August 2021.

**Figure 1:**
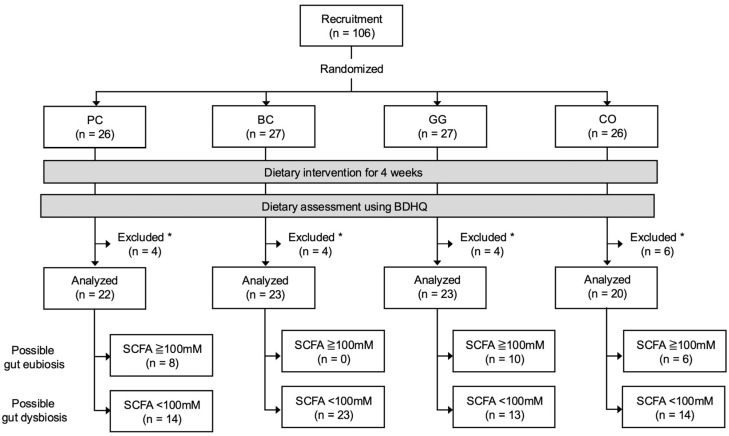
Flow chart of study participants. PC, placebo group; BC, blackcurrant group; GG, guar gum group; CO, combination of blackcurrant and guar gum group; BDHQ, brief dietary history questionnaire; SCFA, short-chain fatty acid. * Participants who did not provide samples or questionnaires due to personal reasons were excluded from downstream analyses.

A total of 106 male rugby players from Setsunan University were selected as participants based on following criteria: (1) healthy male belonging to the Setsunan university’s rugby team; (2) regularly participating in the team’s practices (at least 6 days per week, 3 h per day); and (3) with no previously diagnosed gastrointestinal disorders. The study was conducted in a randomized, double-blinded, placebo-controlled manner. The participants were randomly assigned into 4 groups: placebo group (PC, *n* = 26), blackcurrant group (BC, *n* = 27), guar gum group (GG, *n* = 27), and combination of blackcurrant and guar gum group (CO, *n* = 26) (Figure 1). Randomization was carried out with a permuted block method and group assignment to each block was conducted by a person who was unaware of the study details and backgrounds of the participants (Hiromi Ikeda, PhD at Setsunan University). The key (links between groups and blocks) was kept by H. Ikeda and blinded to all participants and researchers until all analyses were finalized.

All participants were dietary-intervened for 4 weeks as follows. During the experiment, the PC group was supplemented with 6 g/d of maltodextrin as a placebo against PHGG (Sunfiber^®^; Taiyo Kagaku, Yokkaichi, Japan) [23] and 900 mg/d of placebo tablets (3 tablets) consisting of crystalline cellulose and maltitol as placebo blackcurrant extract [24]; the BC group was supplemented with 6 g/d of placebo powder and 900 mg/d of tablets containing 150 mg of blackcurrant extract; the GG group was supplemented with 6 g/d of PHGG powder and 900 mg/d of placebo tablets; and the CO group was supplemented with 6 g/d of PHGG powder and 900 mg/d of blackcurrant extract-containing tablet.

The participants collected their fecal samples with ad hoc scoop and container sets (Sarstedt K.K., Tokyo, Japan) at the beginning (week 0) and the end (week 4) of the experiment. The fecal samples were kept at 4 °C at all times and brought to the laboratory within 24 h after collection. After reception, the fecal samples were stored at −25 °C. Finally, a total of 88 participants (PC, *n* = 22; BC, *n* = 23; GG, *n* = 23; CO, *n* = 20) were included in the analyses in the study, effectively excluding those who did not provide samples or questionnaires due to personal reasons. For an additional subgroup analysis, 64 of 88 participants (PC, *n* = 14; BC, *n* = 23; GG, *n* = 13; CO, *n* = 14) were further identified as potentially having gut dysbiosis based on the concentrations of fecal SCFA at week 0 of the experiment (details in the Section 3).

### 2.2. Dietary Assessment

After the experiment, a survey regarding the dietary habits of participants was conducted using a brief dietary history questionnaire (BDHQ) [25] (Approval Number: 2021-029, approval dates: 15 September 2021). The BDHQ is a self-administered questionnaire that asks the frequency of consumption of foods commonly eaten in Japan so that daily energy and nutrient intake based on an algorithm that incorporates the nutrient composition of each food, derived from the Standard Tables of Food Composition in Japan, can be estimated. The BDHQ typically assesses dietary habits over the previous month. However, in the present study, participants were asked to recall and respond based on their dietary habits over the past three months, including the experiment period. In addition, participants were requested not to consider the blackcurrant and/or PHGG ingested during the experiment period in their response for the BDHQ.

### 2.3. Measurement of Fecal Organic Acid Concentrations

For the measurement of fecal organic acids, including acetate, propionate, iso-butyrate, butyrate, iso-valerate, valerate, succinate, lactate, and formate, 0.3 g of feces was mixed with 600 µL of distilled water and 90 µL of 14% perchloric acid, and centrifuged at 13,000× *g* for 10 min at 4 °C. The supernatants were filtered through 0.45 μm cellulose acetate membrane filters (Cosmonice Filter W, Nakalai Tesque, Kyoto, Japan) and degassed by vacuum. The resulting supernatants were subjected to organic acid measurement using a high-performance liquid chromatography apparatus with an SIL-10 autoinjector (Shimadzu, Kyoto, Japan), as previously described [9].

### 2.4. Analysis of the Fecal Microbiota

Fecal microbial DNA extraction, library preparation, and MiSeq sequencing were conducted as previously described [26]. Briefly, microbial DNA was extracted and purified from 25 mg of feces using the QuickGene DNA Tissue kit SII (KURABO, Osaka, Japan). Microbial DNA was then used to amplify the V3–V4 region of the 16S rRNA gene using the primer sets 341F (5′-CCTACGGGNGGCWGCAG-3′) and 805R (5′-GACTACHVGGGTATCTAATCC-3′) [27]. The PCR steps were conducted according to the following program: initial denaturation at 95 °C for 3 min, followed by 25 cycles of 95 °C for 30 s, 55 °C for 30 s, and 72 °C for 30 s, and a final extension step at 72 °C for 5 min. Amplicons were purified using NucleoFast96 PCR plates (TaKaRa bio, Kusatsu, Japan) and then subjected to a second PCR with unique dual indices primer sets for MiSeq sequencing. The resulting amplicons were purified using a SequalPrep Normalization Plate Kit (Life Technologies, Tokyo, Japan) and AMPure XP beads (Beckman-Coulter, Brea, CA, USA) and pooled, followed by 285 bp paired-end sequencing on the Illumina MiSeq platform (Illumina, San Diego, CA, USA) with MiSeq Reagent Kit v3. Raw sequences were deposited in the NCBI Sequence Read Archive under BioProject ID PRJNA1256317 (available from 1 May 2026).

Data obtained from MiSeq sequencing were analyzed using the QIIME2 version 2022.2 [28]. To construct amplicon sequence variants (ASVs), paired-end reads were denoised using DADA2 via the q2-dada2 plugin [29]. The taxonomic classification of ASVs was carried out using the Naive Bayes classifier via the q2-classifier sklearn plugin against the SILVA 138 99% reference dataset. Singletons and ASVs assigned to mitochondria and chloroplasts were removed from downstream analyses. A phylogenetic tree was generated by SATé-enabled phylogenetic placement (SEPP) [30]. Alpha diversity indices were calculated by QIIME2 by setting the sampling depth at 5000. Outputs from QIIME2 were further analyzed with the R program using the bioconductor packages of Phyloseq [31] and MicrobiotaProcess [32]. Beta diversities were calculated based on weighted and unweighted UniFrac distances.

### 2.5. Statistical Analysis

Fecal organic acid concentrations, microbial alpha-diversity indices, and relative abundances of bacterial taxa in the respective groups were compared between weeks 0 and 4 using a paired *t*-test. In the two-group comparison between participants with gut eubiosis and dysbiosis, fecal microbial alpha-diversity indices and relative abundances of bacterial taxa were compared using Welch’s *t*-test. In the multiple comparison between dietary groups, the statistical differences were first evaluated using a Kruskal–Wallis test and then a Steel–Dwass test. Differences in fecal microbial beta-diversity were analyzed using permutational multivariate analysis of variance (PERMANOVA) with 9999 permutations. *p*-values of <0.05 and <0.10 were considered to be statistically significant and with a tendency to be significant, respectively.

## 3. Results

### 3.1. Characteristics of Participants

A total of 88 participants (PC, *n* = 22; BC, *n* = 23; GG, *n* = 23; CO, *n* = 20) were included in the analyses of the study, excluding those who did not provide samples or questionnaires due to personal reasons (Figure 1). There were no significant differences in the participants’ age, height, weight, and body mass index between groups (Table 1). The macronutrient intake estimated by BDHQ is shown in Table S1.

### 3.2. Effects of Blackcurrant Extract and PHGG Intake on Fecal Organic Acid Concentrations and the Microbiota

Fecal organic acid concentrations measured at weeks 0 and 4 of the experiment are shown in Table 2. During the experiment, total SCFA concentrations did not change in the PC, GG, and CO groups, but significantly increased from week 0 to week 4 in the BC group (*p* < 0.001), although the initial concentrations in the BC group were considerably lower than in the other groups. Regarding each organic acid, a significant decrease in iso-butyrate concentration was observed in the PC group (*p* < 0.05). Significant increases in all SCFAs and formate concentrations were observed in the BC group (*p* < 0.05). During the experiment, the propionate concentration showed a tendency to increase in the CO group (*p* < 0.10).

Comparisons of fecal microbial alpha- and beta-diversities between weeks 0 and 4 are shown in Figure S1. During the experiment, Chao1 and Shannon indices did not change, irrespective of groups (Figure S1A). PCoA plots based on weighted UniFrac distances also did not show significant differences in the bacterial community structures between weeks 0 and 4, irrespective of groups (Figure S1B).

Bacterial taxa that showed statistical differences in the relative abundances between weeks 0 and 4 are shown in Table 3. The relative abundances of 10, 3, 6, and 9 bacterial taxa showed statistically significant changes in PC, BC, GG, and CO groups, respectively. For example, the relative abundance of *Megasphaera* spp. tended to increase (*p* < 0.10) from week 0 to week 4 in BC and GG groups and that of *Bifidobacterium* spp. significantly increased (*p* < 0.05) in the CO group.

### 3.3. Identification of Participants with Gut Dysbiosis

Based on the results from the measurement of organic acids (Section 3.2, Table 2), with the exception of the BC group, the effects of the dietary interventions were less apparent. Increased SCFA concentrations were observed only in the BC group, which encompassed many participants with lower concentrations at week 0. Thus, we speculated that dietary interventions affected more participants with dysbiotic gut environments than with eubiotic gut environments. Therefore, to further evaluate the effects of the dietary interventions, a subgroup analysis focusing on participants with gut dysbiosis was conducted.

To identify the participants with gut dysbiosis, we grouped the participants with total fecal SCFA concentrations > 100 mM (*n* = 24) and <100 mM (*n* = 64) at baseline. This was carried out according to a meta-analysis report by Xu et al. [8], demonstrating that the total fecal SCFA concentrations of ulcerative colitis patients with gut dysbiosis ranged within 35–100 mM. We compared the bacterial diversity and composition between the above two groups and observed typical gut dysbiotic characteristics in participants with total fecal SCFA concentrations < 100 mM (details in Supplementary Text S1; Figure S2; Tables S2 and S3). Based on the results, at least in the present work, participants with total fecal SCFA concentrations > 100 mM and <100 mM were classified as participants with possible gut eubiosis and dysbiosis, respectively. Detailed differences in gut microbiota and dietary habits between these two groups are discussed in the Supplementary Text S1 because it was beyond the scope of the present study.

### 3.4. Subgroup Analysis of the Effects of Blackcurrant Extract and/or PHGG Intake on the Fecal SCFA Concentrations in Participants with Possible Gut Dysbiosis

The fecal organic acid concentrations of the participants with possible gut dysbiosis are shown in Table 4. During the experiment, the total SCFA did not change in the PC group but significantly increased in the BC (*p* < 0.001) and CO (*p* < 0.05) groups, and tended to increase in GG (*p* < 0.10). Regarding each organic acid, significant increases (*p* < 0.05) of all SCFAs and formate concentrations were observed in the BC group, and a tendency of the propionate concentration to increase (*p* < 0.10) was observed in the GG group. In addition, a significant increase in propionate (*p* < 0.05) and a tendency of the acetate concentration to increase (*p* < 0.10) were observed in the CO group.

### 3.5. Subgroup Analysis of the Effects of Blackcurrant Extract and PHGG Intake on the Fecal Microbiota in Participants with Possible Gut Dysbiosis

Results of alpha- and beta-diversity analyses in participants with possible gut dysbiosis are shown in Figure S3. There were no significant differences in any of the diversity analyses between weeks 0 and 4, irrespective of groups.

The relative abundances of each bacterial taxa in the respective groups were compared between weeks 0 and 4 (Table 5). In the PC group, while significant decreases were observed in the relative abundances of the *Escherichia*/*Shigella* group (*p* < 0.05), as well as tendencies to decrease in the abundances of *Blautia* spp. (*p* < 0.10), *Phascolarctobacterium* spp. (*p* < 0.10), and the putative *Clostridium innocuum* group (*p* < 0.10), tendencies to increase in the abundances of *Megamonas* spp (*p* < 0.10) and *Flavonifracter* spp. (*p* < 0.10) were detected. In the BC group, from week 0 to week 4, the relative abundances of *Megasphaera* spp. and *Phascolarctobacterium* spp. tended to increase (*p* < 0.10), while that of *Subdoligranulum* spp. significantly decreased (*p* < 0.05). In the GG group, while the relative abundances of *Faecalibacterium* spp. and *Veillonella* spp. tended to increase (*p* < 0.10), the relative abundance of the *Eubacterium coprostanoligenes* group tended to decrease (*p* < 0.10) and *Tyzzerella* spp. (*p* < 0.05) significantly decreased. In the CO group, from week 0 to week 4, while the relative abundance of unclassified Enterobacteriaceae tended to increase (*p* < 0.10), the relative abundances of unclassified Lachnospiraceae (*p* < 0.10), putative *Ruminococcus torques* group (*p* < 0.1), *Lachnoclostridium* spp. (*p* < 0.10), and *Lachnospira* spp. (*p* < 0.10) tended to decrease, and *Blautia* spp. (*p* < 0.05), *Fusicatenibacter* spp. (*p* < 0.05), the Lachnospiraceae ND 3007 group (*p* < 0.05), and Lachnospiraceae UCG-004 (*p* < 0.05) were observed to decrease.

## 4. Discussion

Our previous study reported that male university rugby players tended to experience gut dysbiosis [9]. In the present study, we evaluated blackcurrant extract and/or PHGG as functional materials to improve the gut environment of male university rugby players. First, we assessed the effects of blackcurrant extract and/or PHGG intake on the gut environment based on the results of all 88 participants. A microbiota analysis showed that dietary interventions did not alter the overall bacterial community structure, but did alter the relative abundance of some bacterial taxa. For example, an increase in *Megasphaera* spp., a beneficial microbe that produces butyrate, was observed in the BC and GG groups. In the CO group, *Bifidobacterium* spp., which is known to be a beneficial, acetate-producing microbe, increased. Although we observed an increased propionate concentration in the CO group, the total fecal SCFA concentration increased only in the BC group but not in the GG and CO groups. Participants in the BC group had considerably lower SCFA concentrations at week 0 compared with those of other groups. Therefore, we speculated that dietary interventions affected participants with a dysbiotic gut environment more than those with a eubiotic gut environment. We then attempted to conduct a subgroup analysis focusing on participants with gut dysbiosis to further evaluate the effects of the dietary interventions.

Gut dysbiosis is not quantitatively defined but is characterized by typical features such as decreased bacterial diversity, increased harmful bacteria, decreased beneficial bacteria, and decreased SCFA concentrations [8,33,34,35]. In the present study, we attempted to identify participants with possible gut dysbiosis based not on alpha-diversity indices but on fecal SCFA concentrations, because there is not an absolute scale; values tend to vary depending on several factors, such as the data analysis pipeline, and hence it is hard to establish a threshold based on published research. Xu et al. [8] reported that the total fecal SCFA concentrations of ulcerative colitis patients with gut dysbiosis ranged within 35–100 mM based on the meta-analysis of 11 studies. Indeed, in the present study, participants with total fecal SCFA < 100 mM also exhibited typical characteristics of gut dysbiosis (see Section 3.3). Hence, at least in the present work, participants with total fecal SCFA concentrations > 100 mM and <100 mM were classified as participants with possible gut eubiosis and dysbiosis, respectively. It should be noted that, in the present study, between participants with eubiotic and dysbiotic guts, there were differences in micronutrient intake, including several vitamins and minerals. The relation of these micronutrients to gut dysbiosis is of interest, and thus further investigation is needed to clarify this relationship.

For participants with possible gut dysbiosis, blackcurrant extract and/or PHGG intake significantly increased fecal SCFA concentrations, regardless of whether they were ingested solely or together. Specifically, the intake of blackcurrant and/or PHGG increased the propionate concentrations. This result suggests that blackcurrant extract and/or PHGG intake have different impacts on the dysbiotic gut microbiota. Because the intervention did not drastically alter the overall gut bacterial community structure, it is possible that blackcurrant extract and/or PHGG affected the metabolic activity of gut microbiota rather than inducing major shifts in microbial composition and increased SCFA concentrations. Further research is needed to clarify the underlying mechanisms.

Below, we discuss the bacterial taxa that changed in participants with possible gut dysbiosis. For those in the BC group, increases in *Megasphaera* spp. and *Phascolarctobacterium* spp. were observed. The former and latter consume lactate and succinate, respectively, and produce acetate and propionate [36,37]. Although the mechanism has not yet been clarified, increases in these bacteria by ingesting plant-derived anthocyanins were in line with previous reports [38]. Increases in these bacteria may be a possible cause of increased fecal acetate and propionate in the BC group. In addition, the accumulation of lactate and succinate is occasionally seen in athletes with gut dysbiosis, and it can lead to gut inflammation and diarrhea [9,16]. Although in the present study we did not observe an accumulation of lactate and succinate, increases in *Megasphaera* spp. and *Phascolarctobacterium* spp. seem to be beneficial for the gut health of athletes.

Similarly, for the participants with possible gut dysbiosis in the GG group, the relative abundance of *Veillonella* spp., which consume lactate and succinate and produce acetate and propionate [37], increased from weeks 0 to 4. In addition, PHGG intake increased the relative abundance of *Faecalibacterium* spp., as reported in a previous work [39]. *Faecalibacterium* spp. have been known to produce acetate and butyrate and have anti-inflammatory properties [40,41]. Numerical increases in acetate (1.26-fold) and butyrate (1.38-fold) were also observed in the GG group, although they were not statistically significant, probably due to large individual differences. Increases in *Veillonella* spp. and *Faecalibacterium* spp. due to PHGG intake can aid in improving gut dysbiosis via SCFA production.

In view of the CO group with possible gut dysbiosis, various uncharacterized Lachnospiraceae decreased. As genera belonging to Lachnospiraceae tend to produce SCFA, this change seems to be in conflict with the increase in fecal SCFA in this group. A detailed link between the taxonomical alteration in the gut microbiota and an increase in SCFA concentrations was beyond the scope of the present study, but, as discussed above, changes in the metabolic activity of the gut microbiota may be a possible mechanism explaining this contradiction. This point should be further investigated in the future using whole-genome shotgun metagenomics or metatranscriptomics techniques.

Regarding the synergistic effect of blackcurrant extract and PHGG, some unique effects that were not observed in the solo intake groups were observed in the combinational intake groups, regardless of gut dysbiosis (see the discussion above). However, when analyzed by two-way ANOVA, the statistical interaction was not confirmed for the beneficial changes observed, such as increases in SCFA concentrations and abundance of *Bifidobacterium* spp. Furthermore, additive effects such as the increases in butyrate, iso-butyrate, valerate, and iso-valerate observed in the BC group, along with increases in beneficial bacteria, were not observed even in the dysbiotic participants in the CO group. Although blackcurrant extract and PHGG are different types of functional ingredient—the former is an anthocyanin and the latter is a dietary fiber—their effects on the gut microbiota are likely similar. Both increase *Bifidobacterium* spp. and/or *Bacteroides* spp. in the human gut [42,43], and thus blackcurrant extract and PHGG may possibly have acted competitively in this study. Different dosages of each ingredient may result in a synergistic effect or an effect that leans more towards one ingredient. Further research is required regarding the combined use of these two ingredients.

Nevertheless, based on the results in this study, it is suggested that the beneficial impact on the gut microbiota of male university rugby players is somewhat expected due to the combinational intake of blackcurrant extract and PHGG; however, these are not necessarily taken together, as the intake of blackcurrant extract or PHGG alone exerted notably beneficial effects on the gut microbiota, comparable to—or even exceeding—those observed with their combined intake. The mechanisms underlying the effects of blackcurrant extract and/or PHGG ingestion are beyond the scope of this study, but, as mentioned above, both ingredients are reported to be fermented by beneficial gut bacteria, and thus prebiotic action would be one of the possible mechanisms [42,43]. Future research incorporating omics-based methodologies (e.g., metagenomics, metabolomics) will be crucial for elucidating the mode of actions for both ingredients.

There are several limitations in the present study. The present findings are derived from a specific cohort, namely male university rugby players in Japan. We believe that these findings may be applicable to other athletes who exhibit gut microbial disorders resulting from similar factors (e.g., endurance runners [16]), but their applicability to more or different populations compared to the current cohort (e.g., healthy non-athlete females) requires further research. Additionally, the effects of longer-term interventions should be investigated to determine whether more sustained changes in gut microbiota and host responses can be observed.

## 5. Conclusions

In the present study, we observed that the intake of blackcurrant extract and/or PHGG increased beneficial gut bacteria such as *Megasphaera* spp. and *Bifidobacterium* spp. In addition, a subgroup analysis showed that the dietary interventions had greater beneficial impacts, including increased beneficial bacteria and SCFA concentrations, on participants with dysbiotic gut environments compared to those with eubiotic gut environments. Although further investigation is needed, blackcurrant extract and PHGG can be used at least as functional materials to improve gut dysbiosis in university rugby players.

## Figures and Tables

**Table 1:** Information of participants included in the analyses.

Item	PC	BC	GG	CO	*p*-Value ^a^
N	22	23	23	20	N/A
Age	19.6 ± 1.4	19.7 ± 1.0	20.0 ± 1.3	20.1 ± 1.3	0.61
Height, cm	173.0 ± 6.0	173.0 ± 6.4	172.7 ± 4.2	172.0 ± 4.0	0.96
Body weight, kg	85.4 ± 12.8	82.2 ± 11.6	84.4 ± 9.6	84.4 ± 11.0	0.83
Body mass index	28.0 ± 3.2	27.6 ± 3.5	28.5 ± 3.6	28.5 ± 3.2	0.71
Team position, N					
Backs	8	10	10	7	N/A
Forwards	14	13	13	13	N/A

PC = Placebo group, BC = blackcurrant group, GG = guar gum group, CO = combination of blackcurrant and guar gum group. ^a^ *p*-values were calculated by the Kruskal–Wallis test.

**Table 2:** Effects of blackcurrant extract and/or PHGG intake on fecal organic acid concentrations.

Item	PC (*n* = 22)		BC (*n* = 23)		GG (*n* = 23)		CO (*n* = 20)	
	0 wk	4 wk	0 wk	4 wk	0 wk	4 wk	0 wk	4 wk
Total SCFA ^a^, mM	95.06 ± 28.28	84.35 ± 32.84	26.11 ± 15.69	85.89 ± 27.11 ***	97.66 ± 43.94	96.01 ± 30.35	86.02 ± 29.71	95.99 ± 31.29
Acetate, mM	59.50 ± 18.42	52.41 ± 22.08	16.77 ± 11.57	54.81 ± 21.03 ***	61.10 ± 27.33	59.65 ± 22.75	57.15 ± 22.70	61.82 ± 22.37
Propionate, mM	21.76 ± 8.70	19.19 ± 9.25	5.34 ± 3.49	18.06 ± 6.53 ***	18.36 ± 11.52	19.44 ± 6.24	16.87 ± 8.46	23.01 ± 13.23 ^#^
Iso-butyrate, mM	2.25 ± 2.02	1.26 ± 1.06 *	0.37 ± 0.33	1.29 ± 1.03 ***	1.48 ± 2.08	1.29 ± 1.06	0.86 ± 0.94	0.76 ± 1.18
Butyrate, mM	8.90 ± 5.55	9.59 ± 6.11	3.08 ± 1.72	9.76 ± 4.84 ***	14.06 ± 8.10	13.34 ± 7.93	10.12 ± 5.46	9.37 ± 4.99
Iso-valerate, mM	1.76 ± 2.01	1.16 ± 1.10	0.34 ± 0.36	0.75 ± 1.20 *	1.37 ± 2.20	1.21 ± 1.01	0.75 ± 0.93	0.72 ± 1.06
Valerate, mM	0.90 ± 1.23	0.74 ± 1.23	0.22 ± 0.35	1.23 ± 0.94 ***	1.29 ± 2.99	1.07 ± 1.58	0.26 ± 0.47	0.30 ± 0.79
Succinate, mM	1.48 ± 2.06	2.08 ± 3.83	0.59 ± 1.09	1.40 ± 6.03	2.75 ± 4.82	3.36 ± 8.69	2.80 ± 3.93	2.74 ± 3.93
Lactate, mM	0.27 ± 0.68	1.01 ± 3.87	0.13 ± 0.42	1.03 ± 1.82	0.58 ± 1.77	0.12 ± 0.32	0.94 ± 3.15	2.52 ± 9.34
Formate, mM	0.56 ± 1.94	0.60 ± 1.37	0.10 ± 0.37	0.98 ± 1.57 *	0.08 ± 0.16	0.34 ± 0.92	0.16 ± 0.36	0.41 ± 0.95

PC = Placebo group, BC = blackcurrant group, GG = guar gum group, CO = combination of blackcurrant and guar gum group. Values (means ± SD) were compared between 0 and 4 wks in respective groups using a paired *t*-test (*** *p* < 0.001, * *p* < 0.05, ^#^ *p* < 0.10). ^a^ Total SCFA means the sum of acetate, propionate, iso-butyrate, butyrate, iso-valerate, and valerate.

**Table 3:** Effects of blackcurrant extract and/or PHGG intake on fecal bacterial composition. Symbols “↑” and “↓” mean increase and decrease, respectively.

Group	Taxa ^a^	0 wk	4 wk	0 to 4 wk	*p*-Value ^b^
PC	*Blautia* spp.	13.25 ± 10.62	8.91 ± 6.18	↓	0.033
	*Faecalibacterium* spp.	5.75 ± 6.53	8.63 ± 7.89	↑	0.047
	*Megamonas* spp.	5.19 ± 11.91	9.56 ± 18.36	↑	0.031
	[*Ruminococcus*] *gnavus* group	4.59 ± 6.65	3.25 ± 4.56	↓	0.035
	*Phascolarctobacterium* spp.	1.50 ± 2.33	0.83 ± 2.03	↓	0.013
	*Dorea* spp.	1.31 ± 2.18	0.54 ± 1.16	↓	0.034
	Unclassified Oscillospiraceae	0.23 ± 0.43	0.08 ± 0.16	↓	0.032
	[*Clostridium*] *innocuum* group	0.18 ± 0.37	0.11 ± 0.30	↓	0.007
	[*Ruminococcus*] *gauvreauii* group	0.16 ± 0.47	0.33 ± 0.82	↑	0.071
	*Faecalitalea* spp.	0.10 ± 0.18	0.04 ± 0.09	↓	0.029
BC	*Subdoligranulum* spp.	3.37 ± 4.63	2.13 ± 2.69	↓	0.040
	*Megasphaera* spp.	1.85 ± 5.74	3.73 ± 9.57	↑	0.092
	*Phascolarctobacterium* spp.	0.55 ± 0.88	1.28 ± 1.93	↑	0.088
GG	*Streptococcus* spp.	4.36 ± 4.90	2.75 ± 3.01	↓	0.076
	Unclassified Lachnospiraceae	3.23 ± 2.82	2.08 ± 2.22	↓	0.010
	*Megasphaera* spp.	1.71 ± 4.19	3.04 ± 5.49	↑	0.087
	*Parabacteroides* spp.	0.61 ± 1.01	1.14 ± 1.77	↑	0.095
	[*Eubacterium*] *coprostanoligenes* group	0.35 ± 0.76	0.08 ± 0.24	↓	0.033
	*Tyzzerella* spp.	0.14 ± 0.23	0.04 ± 0.09	↓	0.036
CO	*Blautia* spp.	15.28 ± 7.31	10.44 ± 7.45	↓	0.009
	*Bifidobacterium* spp.	6.95 ± 5.88	14.30 ± 14.06	↑	0.036
	Unclassified Lachnospiraceae	4.07 ± 4.14	2.39 ± 2.40	↓	0.047
	[*Ruminococcus*] *torques* group	2.89 ± 3.67	1.68 ± 1.84	↓	0.045
	*Collinsella* spp.	2.21 ± 1.99	4.84 ± 4.96	↑	0.009
	*Lachnospira* spp.	0.27 ± 0.64	0.03 ± 0.06	↓	0.087
	Lachnospiraceae ND3007 group	0.26 ± 0.36	0.09 ± 0.15	↓	0.033
	Lachnospiraceae UCG-004	0.17 ± 0.27	0.07 ± 0.15	↓	0.013
	Unclassified Enterobacteriaceae	0.11 ± 0.18	0.63 ± 1.15	↑	0.050

PC = Placebo group, BC = blackcurrant group, GG = guar gum group, CO = combination of blackcurrant and guar gum group. Values mean the relative abundances (% of total bacteria) and are shown as the means ± SD. ^a^ Bacterial taxa with a mean relative abundance > 0.1% in the dataset showing statistical significance or tendency to be significant were listed. ^b^ *p*-values were calculated by the paired *t*-test.

**Table 4:** Subgroup analysis of the effect of blackcurrant extract and/or PHGG intake on fecal organic acid concentrations, focusing on participants with possible gut dysbiosis.

Item	PC (Gut Dysbiosis) (*n* = 14)		BC (Gut Dysbiosis) (*n* = 23)		GG (Gut Dysbiosis) (*n* = 13)		CO (Gut Dysbiosis) (*n* = 14)	
	0 wk	4 wk	0 wk	4 wk	0 wk	4 wk	0 wk	4 wk
Total SCFA ^a^, mM	76.83 ± 14.13	79.84 ± 31.94	26.11 ± 15.69	85.89 ± 27.11 ***	69.13 ± 26.61	91.05 ± 24.73 ^#^	69.78 ± 16.41	95.83 ± 36.43 *
Acetate, mM	48.39 ± 9.59	50.37 ± 20.16	16.77 ± 11.57	54.81 ± 21.03 ***	44.97 ± 17.19	56.99 ± 16.53	45.58 ± 11.31	60.58 ± 25.09 ^#^
Propionate, mM	17.69 ± 6.20	17.93 ± 10.66	5.34 ± 3.49	18.06 ± 6.53 ***	11.97 ± 6.68	18.18 ± 6.51 ^#^	13.91 ± 6.25	24.30 ± 14.50 *
Butyrate, mM	6.70 ± 3.69	8.42 ± 6.18	3.08 ± 1.72	9.76 ± 4.84 ***	9.61 ± 4.35	12.86 ± 7.79	8.55 ± 4.21	8.89 ± 4.48
Iso-butyrate, mM	2.00 ± 2.18	1.28 ± 1.21 ^#^	0.37 ± 0.33	1.29 ± 1.03 ***	1.11 ± 1.32	1.14 ± 1.01	0.77 ± 0.87	0.82 ± 1.30
Valerate, mM	0.70 ± 1.25	0.62 ± 0.89	0.22 ± 0.35	0.75 ± 1.20 *	0.59 ± 0.91	0.82 ± 1.32	0.28 ± 0.50	0.42 ± 0.93
Iso-Valerate, mM	1.36 ± 1.78	1.23 ± 1.28	0.34 ± 0.36	1.23 ± 0.94 ***	0.88 ± 1.25	1.06 ± 0.88	0.70 ± 0.78	0.82 ± 1.20
Lactate, mM	0.28 ± 0.76	1.42 ± 4.82	0.13 ± 0.42	1.40 ± 6.03	0.96 ± 2.31	0.20 ± 0.41	0.30 ± 0.76	0.44 ± 0.85
Succinate, mM	1.42 ± 1.94	2.72 ± 4.69	0.59 ± 1.09	1.03 ± 1.82	3.63 ± 5.87	4.80 ± 11.33	2.64 ± 3.83	2.99 ± 4.33
Formate, mM	0.85 ± 2.41	0.66 ± 1.65	0.10 ± 0.37	0.98 ± 1.57 *	0.12 ± 0.21	0.55 ± 1.20	0.18 ± 0.41	0.52 ± 1.12

PC = Placebo group, BC = blackcurrant group, GG = guar gum group, CO = combination of blackcurrant and guar gum group. Values (means ± SD) were compared between 0 and 4 wks in respective groups using a paired *t*-test (*** *p* < 0.001, * *p* < 0.05, ^#^ *p* < 0.10). ^a^ Total SCFA means the sum of acetate, propionate, iso-butyrate, butyrate, iso-valerate, and valerate.

**Table 5:** Subgroup analysis of the effects of blackcurrant extract and/or PHGG intake on fecal microbiota, focusing on participants with possible gut dysbiosis. Symbols “↑” and “↓” mean increase and decrease, respectively.

Group	Taxa ^a^	0 wk	4 wk	0 to 4 wk	*p*-Value ^b^
PC (gut dysbiosis)	*Blautia* spp.	14.07 ± 11.67	8.82 ± 5.93	↓	0.060
	*Megamonas* spp.	7.59 ± 14.50	12.65 ± 22.01	↑	0.090
	*Escherichia–Shigella* group	3.33 ± 4.63	1.24 ± 2.02	↓	0.044
	*Phascolarctobacterium* spp.	1.65 ± 2.78	1.05 ± 2.46	↓	0.081
	*Flavonifractor* spp.	0.13 ± 0.15	0.25 ± 0.25	↑	0.058
	[*Clostridium*] *innocuum* group	0.19 ± 0.46	0.14 ± 0.38	↓	0.065
BC (gut dysbiosis)	*Subdoligranulum* spp.	3.37 ± 4.63	2.13 ± 2.69	↓	0.040
	*Megasphaera* spp.	1.85 ± 5.74	3.73 ± 9.57	↑	0.092
	*Phascolarctobacterium* spp.	0.55 ± 0.88	1.28 ± 1.93	↑	0.088
GG (gut dysbiosis)	*Faecalibacterium* spp.	6.53 ± 7.33	8.41 ± 8.10	↑	0.052
	*Veillonella* spp.	5.95 ± 7.45	8.20 ± 8.70	↑	0.099
	[*Eubacterium*] *coprostanoligenes* group	0.46 ± 0.91	0.08 ± 0.29	↓	0.070
	*Tyzzerella* spp.	0.22 ± 0.26	0.07 ± 0.11	↓	0.047
CO (gut dysbiosis)	*Blautia* spp.	16.11 ± 7.74	10.54 ± 7.01	↓	0.027
	Unclassified Lachnospiraceae	4.25 ± 4.23	2.62 ± 2.76	↓	0.077
	[*Ruminococcus*] torques group	3.65 ± 4.04	2.04 ± 1.89	↓	0.062
	*Fusicatenibacter* spp.	3.65 ± 3.19	1.67 ± 1.95	↓	0.024
	*Lachnoclostridium* spp.	0.93 ± 1.12	0.40 ± 0.38	↓	0.093
	Unclassified Enterobacteriaceae	0.09 ± 0.16	0.72 ± 1.28	↑	0.074
	*Lachnospira* spp.	0.16 ± 0.27	0.02 ± 0.05	↓	0.071
	Lachnospiraceae ND3007 group	0.31 ± 0.40	0.07 ± 0.11	↓	0.033
	Lachnospiraceae UCG-004	0.09 ± 0.17	0.04 ± 0.13	↓	0.035

PC = Placebo group, BC = blackcurrant group, GG = guar gum group, CO = combination of blackcurrant and guar gum group. Values mean the relative abundances (% of total bacteria) and are shown as mean ± SD. ^a^ Bacterial taxa with a mean relative abundance > 0.1% in the dataset showing statistical significance or a tendency to be significant are listed. ^b^ *p*-values were calculated by the paired *t*-test.

## Data Availability

Raw sequences have been deposited in the DDBJ Sequence Read Archive under the BioProject ID PRJNA1256317 (available from 1 May 2026).

## Supplementary Materials

The following supporting information can be downloaded at https://www.mdpi.com/article/10.3390/microorganisms13071561/s1: Table S1: Macronutrient intake of the university rugby players in the present study. Figure S1: Effects of blackcurrant extract and PHGG intake on the alpha- and beta-diversities of the fecal microbiota. Figure S2: Comparison of the alpha- and beta-diversities between participants with total fecal SCFA concentrations > 100 mM and <100 mM. Table S2. Bacterial taxa showing statistical differences in the relative abundances between participants with total fecal SCFA concentrations > 100 mM and <100 mM. Table S3: Comparison of dietary intake between participants with total fecal SCFA concentrations > 100 mM and <100 mM. Text S1: Results and Discussion of the comparison of the gut environment and dietary intake between participants with total fecal SCFA concentrations > 100 mM and <100 mM. Figure S3: Subgroup analysis for the effect of blackcurrant extract and PHGG intake on the alpha- and beta- diversities of the fecal microbiota, focusing on participants with possible gut dysbiosis.
