# Supplementary material for: Effects of Blackcurrant Extract and Partially Hydrolyzed Guar Gum Intake on Gut Dysbiosis in Male University Rugby Players
Source: Microorganisms. 2025 Jul 2;13(7):1561. doi: 10.3390/microorganisms13071561 (PMC12299167; doi:10.3390/microorganisms13071561)
Supplement: Supplementary file 1 [file microorganisms-13-01561-s001.zip › microorganisms-3644076-supplementary.pdf]

Supplementary information for

Effects of blackcurrant extract and partially hydrolyzed guar gum intake on gut dysbiosis of university male rugby players

Hiroto Miura, Machi Oda, Kanako Abe, Hiromi Ikeda, Mami Fujibayashi, Naoko Oda, Tomohiro, Segawa, Aya Abe, Natsumi Ueta, Takamitsu Tsukahara, Tomohisa Takagi, Yuji Naito, Ryo Inoue

Correspondence to: ryo.inoue@setsunan.ac.jp (Ryo Inoue)

File contents (order of appearance in main text):

Table S1. Macronutrient intake of the university rugby players in the present study

Figure S1. Effects of blackcurrant extract and PHGG intake on the alpha- and beta-diversities of the fecal microbiota.

Figure S2. Comparison of the alpha- and beta-diversities between participants with total fecal SCFA concentrations >100 mM and <100 mM.

Table S2. Bacterial taxa showing statistical differences in the relative abundances between participants with total fecal SCFA concentrations >100 mM and <100 mM.

Table S3. Comparison of dietary intake between participants with total fecal SCFA concentrations >100 mM and <100 mM.

Text S1. Results and discussion of the comparison of the gut environment and dietary intake between participants with total fecal SCFA concentrations >100 mM and <100 mM

Figure S3. Subgroup analysis for the effect of blackcurrant extract and PHGG intake on the alpha- and beta- diversities of the fecal microbiota, focusing on participants with possible gut dysbiosis

Table S1. Macronutrient intake of the university rugby players in the present study [*1]

Items	PC	BC	GG	CO	*P*-value [*2]
Energy, kcal/d	2944 (2403–3290) [ab]	3291 (2583–3919) [a]	2663 (1949–3037) [b]	3401 (2708–4158) [a]	0.009**
Energy-adjusted intake [*3], g/d					
Protein	34.6 (29.8–37.1)	32.0 (28.4–35.1)	33.0 (30.3–37.6)	34.3 (30.6–40.7)	0.329
Fat	27.3 (24.8–33.7)	25.9 (23.8–29.9)	29.4 (26.6–32.1)	30.0 (27.6–35.7)	0.166
Saturated fat	7.7 (6.8–8.9) [ab]	6.3 (5.4–7.6) [b]	7.8 (6.4–8.3) [ab]	8.3 (6.7–10.4) [a]	0.038*
Monounsaturated fat	8.4 (7.6–10.2)	8.1 (6.8–10.3)	8.9 (7.9–10.3)	9.3 (8.2–10.8)	0.467
Polyunsaturated fat	5.5 (4.8–6.6)	5.8 (4.8–6.8)	5.9 (5.2–7.4)	6.1 (5.4–6.5)	0.587
Carbohydrate	149.3 (132.7–156.4)	153.0 (141.2–161.3)	142.9 (133.5–155.6)	141.1 (122.9–151.7)	0.151
Fiber	4.9 (4.3–5.2)	4.8 (4.2–5.2)	5.0 (4.3–5.7)	4.6 (4.1–5.6)	0.921

PC = Placebo group, BC = Blackcurrant group, GG = Guan gum group, CO = combination of blackcurrant and guar gum group

[*1] Values and numbers in brackets represent the median and interquartile range, respectively.

[*2] *P*-values were calculated using the Kruskal-Wallis test, and when $P < 0.05$, the Steel-Dwass test was carried out for pairwise comparisons between groups (a, b: $P < 0.05$).

[*3] The intake of each nutrient was normalized by 1000 kcal of total energy intake

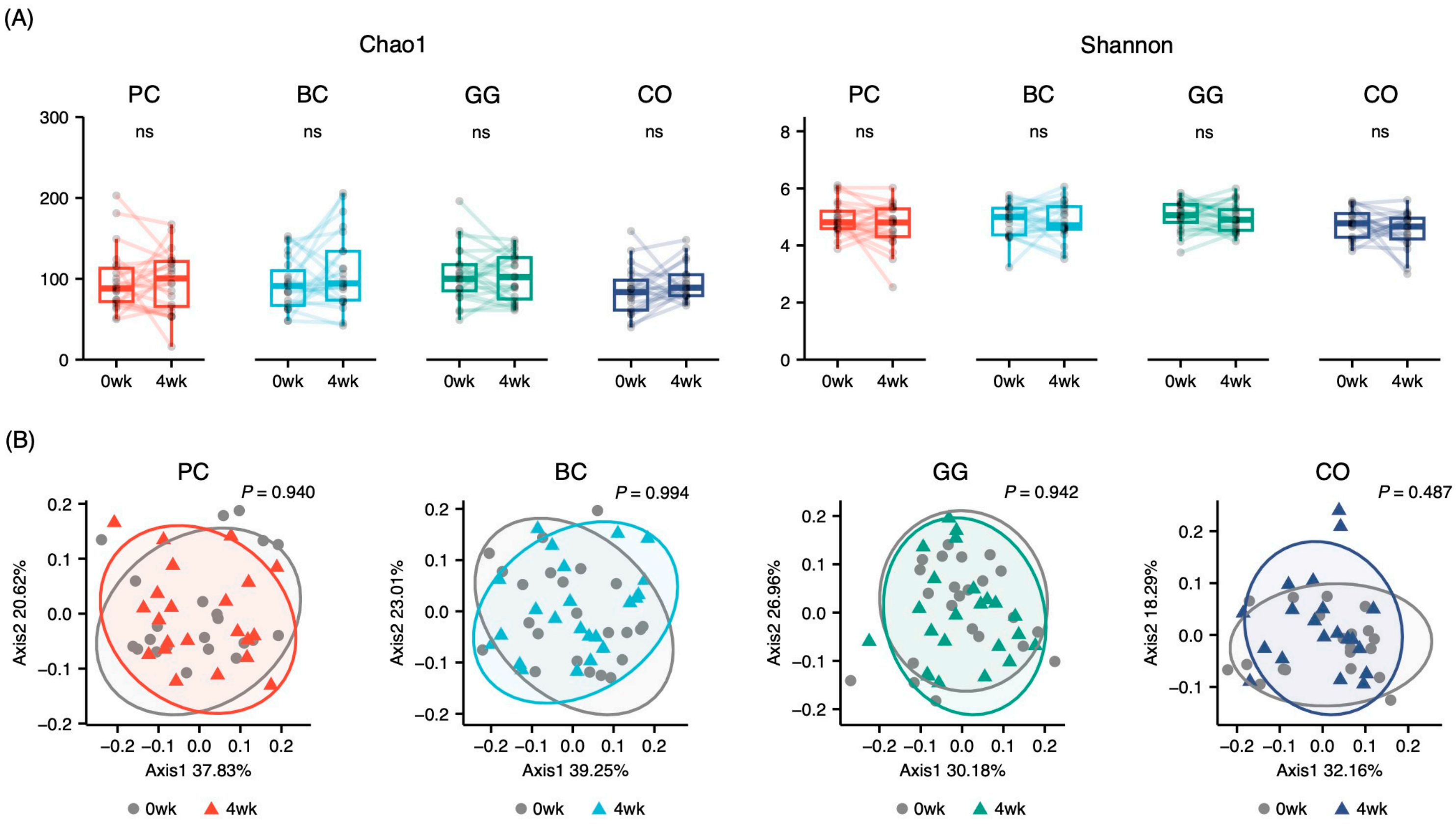

Figure S1. Effects of blackcurrant extract and PHGG intake on the alpha- and beta-diversities of the fecal microbiota. PC = Placebo group, BC = Blackcurrant group, GG = Guan gum group, CO = combination of blackcurrant and guar gum group. (A) Chao1 and Shannon indices of

fecal microbiotas of the respective groups were compared between weeks 0 and 4 with a paired *t*-test (ns, not significant). Dots connected by lines mean individual participants. (B) PCoA plots of the fecal microbiota based on weighted UniFrac distances. Gray and colored dots denote individual participants at weeks 0 and 4, respectively. Ellipses enclosing clusters mean 90% confidence interval. The differences between weeks 0 and 4 were evaluated by PERMANOVA (permutation = 9999).

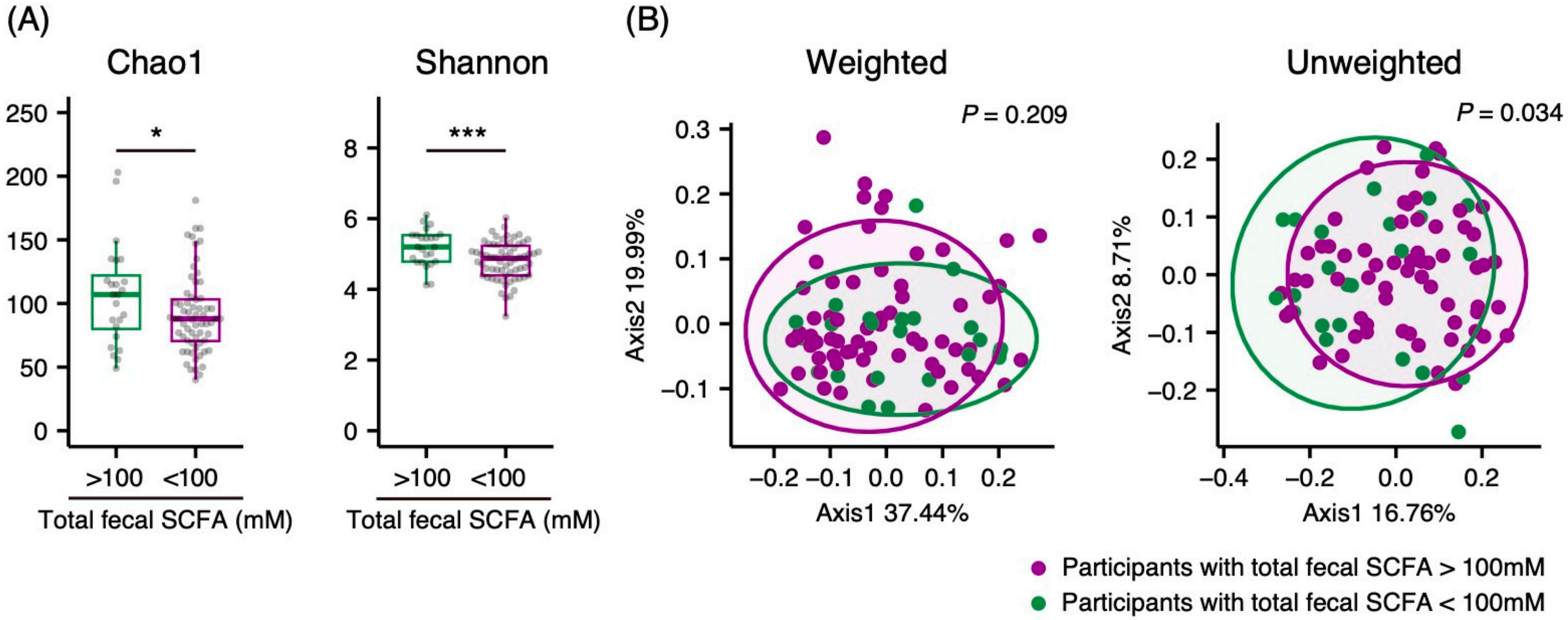

Figure S2. Comparison of the alpha- and beta-diversities between participants with total fecal SCFA concentrations >100 mM and <100 mM. (A) Chao1 and Shannon indices of the fecal microbiotas were compared between groups using Welch's *t*-test (* $P < 0.05$; *** $P < 0.001$). (B) PCoA plots of fecal microbiota based on weighted and unweighted UniFrac distances. Green and purple dots denote individual participants with total fecal SCFA concentrations >100 mM and <100 mM, respectively. Ellipses enclosing clusters mean 90% confidence interval. The differences between weeks 0 and 4 were evaluated by PERMANOVA (permutation = 9999).

Table S2. Bacterial taxa showing statistical differences in relative abundances between participants with total fecal SCFA concentrations >100 mM and <100 mM.

Taxa [*1]	Total fecal SCFA concentration		P-value [*2]
	>100 mM (relatively better)	<100 mM (potential gut dysbiosis)	
Faecalibacterium spp.	11.12 ± 8.94	7.50 ± 7.86	0.089
[Ruminococcus] torques group	1.30 ± 1.68	3.39 ± 5.15	0.005
Escherichia/Shigella group	0.68 ± 1.25	2.43 ± 5.63	0.022
Unclassified Ruminococcaceae	1.05 ± 2.15	0.18 ± 0.54	0.062
Erysipelatoclostridium spp.	0.20 ± 0.47	0.46 ± 0.68	0.051
Holdemanella spp.	N.D.	0.50 ± 2.02	0.051
Lachnospiraceae NK4A136 group	0.14 ± 0.27	0.43 ± 1.26	0.081
Parasutterella spp.	0.57 ± 1.10	0.17 ± 0.41	0.098
Turicibacter spp.	0.04 ± 0.10	0.19 ± 0.57	0.053
Sellimonas spp.	0.21 ± 0.35	0.08 ± 0.19	0.094

Values mean the relative abundances (% of total reads) and are shown as the means ± SD

N.D., Not detected

[*1] Bacterial taxa with a mean relative abundance >0.1% are listed.

[*2] *P*-values were calculated by Welch's *t*-test.

Text S1. Results and Discussion of the comparison of gut environments and dietary intake between participants with total fecal SCFA concentrations >100 mM and <100 mM

We compared the fecal bacterial diversity and composition between participants with total fecal SCFA concentrations >100 mM (n = 24) and <100 mM (n = 64). The former had significantly higher ($P < 0.05$) alpha diversity indices including Chao1 and Shannon than the latter (Figure S2A). Reduced gut bacterial diversity is recognized as a typical phenomenon in gut dysbiosis (DeGrottola et al., 2016; Levy et al., 2017). Bacterial community structures showed significant differences ($P < 0.05$) between the above two groups based on PCoA plots with UniFrac distances (Figure S2B). Regarding the bacterial composition, 10 bacterial taxa showed statistical differences between participants with total fecal SCFA concentrations >100 mM and <100 mM (Table S2). For example, participants with total fecal SCFA concentrations <100 mM had lower ($P < 0.10$) abundances of beneficial bacteria *Faecalibacterium* spp. and significant higher ($P < 0.05$) abundance of harmful *Esherichia*/*Shigera* group. Changes in these bacterial abundances are frequently observed in patients of IBD with gut dysbiosis (DeGrottola et al., 2016; Levy et al., 2017). Based on these, at least in the present work, participants with total fecal SCFA concentrations >100 mM and <100 mM were classified as participants with possible gut eubiosys and dysbiosis, respectively.

Nutrient intake was also compared between participants with possible gut eubiosis and dysbiosis (Table S3). Although no significant differences were observed in macronutrient intake, participants with potential gut dysbiosis had a lower intake of vitamin B2 ($P < 0.05$) and a tendency to have a lower daily intake of potassium ($P < 0.05$), magnesium ($P < 0.10$), iron ($P < 0.10$), vitamin K ($P < 0.10$), folate (vitamin B5) ($P < 0.10$), and pantothenic acid (vitamin B9) ($P < 0.01$), when compared with participants with a eubiotic gut environment. There have been studies reporting that amounts of vitamins and minerals intake affect the gut microbial composition and gut immune/barrier function (Pham, et al., 2021; Ellis et al., 2021; Wu et al., 2022). Our results also seem to support the importance of the intake of these micronutrients to maintain the gut health.

Table S3. Comparison of dietary intake between participants with total fecal SCFA concentrations >100 mM and <100 mM

Items [*1]	Total fecal SCFA concentration		*P*-value [*2]
	>100 mM (relatively better)	<100 mM (potential gut dysbiosis)	
Energy, kcal/d	2822.6 ± 828.8	3133.8 ± 904.6	0.133
Weight, g	1098.9 ± 177.1	1055.9 ± 209.2	0.340
Water, g	881.3 ± 175.6	839.2 ± 208.5	0.347
Protein, g	35.5 ± 5.3	33.7 ± 6.3	0.183
Animal Protein, g	20.0 ± 5.6	18.5 ± 6.8	0.303
Vegetable Protein, g	15.4 ± 2.8	15.1 ± 2.1	0.625
Fat, g	28.7 ± 5.0	28.7 ± 5.9	0.988
Animal Fat, g	11.7 ± 3.7	11.7 ± 3.7	0.970
Vegetable Fat, g	15.8 ± 3.3	15.9 ± 3.5	0.897
Carbohydrate, g	144.7 ± 14.4	145.8 ± 18.2	0.766
Ash, g	8.4 ± 1.3	8.2 ± 1.7	0.604
Sodium, mg	1975.5 ± 375.6	2048.7 ± 416.7	0.434
**Potassium, mg**	**1105.2 ± 258.3**	**971.6 ± 261.3**	**0.037** [*]
Calcium, mg	319.2 ± 108.3	281.8 ± 112.9	0.161
**Magnesium, mg**	**122.6 ± 22.3**	**113.1 ± 22.6**	**0.083** [#]
Phosphorus, mg	540.2 ± 100.5	510.5 ± 109.6	0.234
**Iron, mg**	**4.2 ± 0.9**	**3.8 ± 0.9**	**0.054** [#]
Zinc, mg	4.3 ± 0.6	4.3 ± 0.5	0.543
Copper, mg	0.6 ± 0.1	0.6 ± 0.1	0.219
Manganese, mg	1.7 ± 0.6	1.8 ± 0.5	0.670
Retinol, μg	288.0 ± 376.5	202.0 ± 99.5	0.281
Beta-carotene Equivalent, μg	1291.0 ± 843.3	1149.2 ± 763.2	0.476
Retinol Equivalent, μg	397.4 ± 383.7	299.3 ± 124.2	0.230
Vitamin D, μg	11.6 ± 4.6	10.5 ± 5.4	0.347
Alpha-tocopherol, mg	3.7 ± 0.8	3.6 ± 0.9	0.718
**Vitamin K, μg**	**163.3 ± 103.5**	**121.1 ± 64.3**	**0.072** [#]
Vitamin B1 (Thiamine), mg	0.4 ± 0.1	0.4 ± 0.1	0.321
**Vitamin B2 (Riboflavin), mg**	**0.8 ± 0.2**	**0.7 ± 0.2**	**0.015** [*]
Niacin, mg	8.0 ± 1.7	7.6 ± 1.8	0.305

Vitamin B6, mg	0.6 ± 0.1	0.6 ± 0.1	0.134
Vitamin B12, μg	3.4 ± 1.7	3.3 ± 1.7	0.824
**Folate, μg**	**171.1 ± 60.4**	**145.9 ± 47.2**	**0.074** [#]
**Pantothenic Acid, mg**	**3.9 ± 0.6**	**3.4 ± 0.7**	**0.004** [**]
Vitamin C, mg	54.8 ± 21.2	48.0 ± 18.3	0.170
Saturated Fatty Acids, g	7.6 ± 2.1	7.7 ± 2.4	0.877
Monounsaturated Fatty Acids, g	8.7 ± 1.7	9.1 ± 2.1	0.458
Polyunsaturated Fatty Acids, g	5.9 ± 1.3	5.9 ± 1.3	0.938
Cholesterol, mg	188.0 ± 62.4	162.1 ± 69.8	0.101
Water-soluble Dietary Fiber, g	1.2 ± 0.5	1.1 ± 0.4	0.380
Insoluble Dietary Fiber, g	3.7 ± 1.0	3.5 ± 0.8	0.515
Total Dietary Fiber, g	5.2 ± 1.5	4.9 ± 1.2	0.445
Salt (sodium chloride equivalent), g	5.0 ± 0.9	5.2 ± 1.1	0.443
Sucrose, g	3.5 ± 2.7	3.3 ± 2.2	0.632
Daidzein, mg	7.1 ± 5.6	5.2 ± 3.3	0.121
Genistein, mg	11.9 ± 9.2	8.7 ± 5.6	0.120
n-3 Polyunsaturated Fatty Acids (Omega-3), g	1.0 ± 0.2	1.1 ± 0.3	0.686
n-6 Polyunsaturated Fatty Acids (Omega-6), g	4.9 ± 1.1	5.0 ± 1.0	0.990
Butyric Acid (C4:0 Saturated), mg	126.1 ± 84.8	131.9 ± 92.1	0.782
Caproic Acid (C6:0 Saturated), mg	79.6 ± 53.7	83.9 ± 60.1	0.745
Caprylic Acid (C8:0 Saturated), mg	64.1 ± 38.3	70.5 ± 53.7	0.537
Capric Acid (C10:0 Saturated), mg	119.6 ± 71.7	126.0 ± 87.6	0.730
Caproleic Acid (C10:1 Monounsaturated), mg	10.7 ± 6.9	11.1 ± 7.9	0.797
Lauric Acid (C12:0 Saturated), mg	220.2 ± 129.1	247.5 ± 199.3	0.454
Myristic Acid (C14:0 Saturated), mg	598.1 ± 274.5	630.1 ± 323.2	0.646
Myristoleic Acid (C14:1 Monounsaturated), mg	53.9 ± 27.0	56.7 ± 27.7	0.670
Pentadecanoic Acid (C15:0 Saturated), mg	58.4 ± 28.1	60.7 ± 30.1	0.740
Pentadecenoic Acid (C15:1 Monounsaturated), mg	0.2 ± 0.2	0.2 ± 0.2	0.850
Palmitic Acid (C16:0 Saturated), mg	4374.3 ± 964.3	4408.8 ± 1145.5	0.888

Palmitoleic Acid (C16:1 Monounsaturated), mg	381.5 ± 96.9	374.8 ± 109.3	0.784
16:3 Fatty Acid, mg	4.2 ± 2.4	3.6 ± 2.1	0.325
Margaric Acid (C17:0 Saturated), mg	62.1 ± 19.5	63.9 ± 21.0	0.711
Margaroleic Acid (C17:1 Monounsaturated), mg	44.1 ± 16.5	47.6 ± 16.0	0.371
Stearic Acid (C18:0 Saturated), mg	1768.3 ± 539.0	1728.4 ± 509.4	0.755
Oleic Acid (C18:1 Monounsaturated), mg	8052.1 ± 1560.6	8349.5 ± 1929.3	0.460
Linoleic Acid (C18:2n-6), mg	4815.5 ± 1095.8	4828.3 ± 1011.5	0.960
Alpha-linolenic Acid (C18:3n-3), mg	726.1 ± 183.8	749.2 ± 193.6	0.608
Gamma-linolenic Acid (C18:3n-6), mg	2.5 ± 1.4	2.7 ± 2.0	0.599
Stearidonic Acid (C18:4n-3), mg	21.9 ± 11.9	23.8 ± 15.9	0.556
Arachidic Acid (C20:0 Saturated), mg	71.7 ± 16.7	74.9 ± 17.9	0.433
Eicosenoic Acid (C20:1 Monounsaturated), mg	172.2 ± 43.9	188.0 ± 73.0	0.220
Eicosadienoic Acid (C20:2n-6), mg	19.9 ± 5.8	20.3 ± 6.6	0.779
Dihomo-gamma-linolenic Acid (C20:3n-6), mg	14.5 ± 3.5	13.5 ± 4.7	0.275
Eicosapentaenoic Acid (EPA, C20:5n-3), mg	8.8 ± 4.4	9.3 ± 6.0	0.648
Arachidonic Acid (C20:4n-6), mg	80.2 ± 26.1	70.9 ± 27.3	0.149
Eicosapentaenoic Acid (EPA, C20:5n-3), mg	88.1 ± 48.5	90.7 ± 60.4	0.837
Behenic Acid (C22:0 Saturated), mg	35.5 ± 8.7	36.9 ± 9.0	0.525
Erucic Acid (C22:1 Monounsaturated), mg	91.8 ± 44.4	102.4 ± 73.7	0.414
Docosapentaenoic Acid (DPA, C22:5n-3), mg	28.6 ± 12.5	29.0 ± 16.2	0.899
Docosapentaenoic Acid (C22:5n-6), mg	2.7 ± 1.0	2.7 ± 1.6	0.952
Docosahexaenoic Acid (DHA, C22:6n-3), mg	166.0 ± 76.8	162.6 ± 94.4	0.864
Lignoceric Acid (C24:0 Saturated), mg	16.0 ± 3.6	17.5 ± 3.7	0.109
Nervonic Acid (C24:1 Monounsaturated), mg	15.0 ± 6.6	16.0 ± 8.3	0.550
α-Carotene, μg	91.0 ± 64.1	111.3 ± 97.4	0.262
β-Carotene, μg	1153.9 ± 839.8	1016.4 ± 712.5	0.481

Cryptoxanthin, μg	163.5 ± 109.0	125.5 ± 113.8	0.157
β-Tocopherol, mg	0.2 ± 0.1	0.2 ± 0.1	0.996
γ-Tocopherol, mg	7.9 ± 1.7	7.8 ± 2.0	0.791
δ-Tocopherol, mg	2.0 ± 0.5	1.9 ± 0.5	0.373
Heptanoic Acid (C7:0 Saturated), mg	0.5 ± 0.5	0.4 ± 0.5	0.494
Tridecanoic Acid (C13:0 Saturated), mg	1.4 ± 1.4	1.2 ± 1.5	0.496
Iso-Pentadecanoic Acid (iso-C15:0 Saturated), mg	18.9 ± 12.9	19.7 ± 13.9	0.806
Iso-Palmitic Acid (iso-C16:0 Saturated), mg	8.5 ± 5.7	8.8 ± 6.4	0.830
Iso-Margaric Acid (iso-C17:0 Saturated), mg	18.6 ± 12.5	19.5 ± 13.7	0.770
16:2 Fatty Acid, mg	3.6 ± 1.7	3.7 ± 2.3	0.822
16:4 Fatty Acid, mg	3.0 ± 1.5	3.0 ± 2.1	0.997
21:5n-3 Fatty Acid, mg	2.7 ± 1.4	2.8 ± 2.0	0.863
22:4n-6 Fatty Acid, mg	3.0 ± 1.2	2.6 ± 1.3	0.168

[*1] Nutrient intake is adjusted and expressed as an amount per 1000 kcal of daily energy intake.

[*2] *P*-values were calculated by Welch's *t*-test ([**] $P < 0.01$, [*] $P < 0.05$, [#] $P < 0.10$).

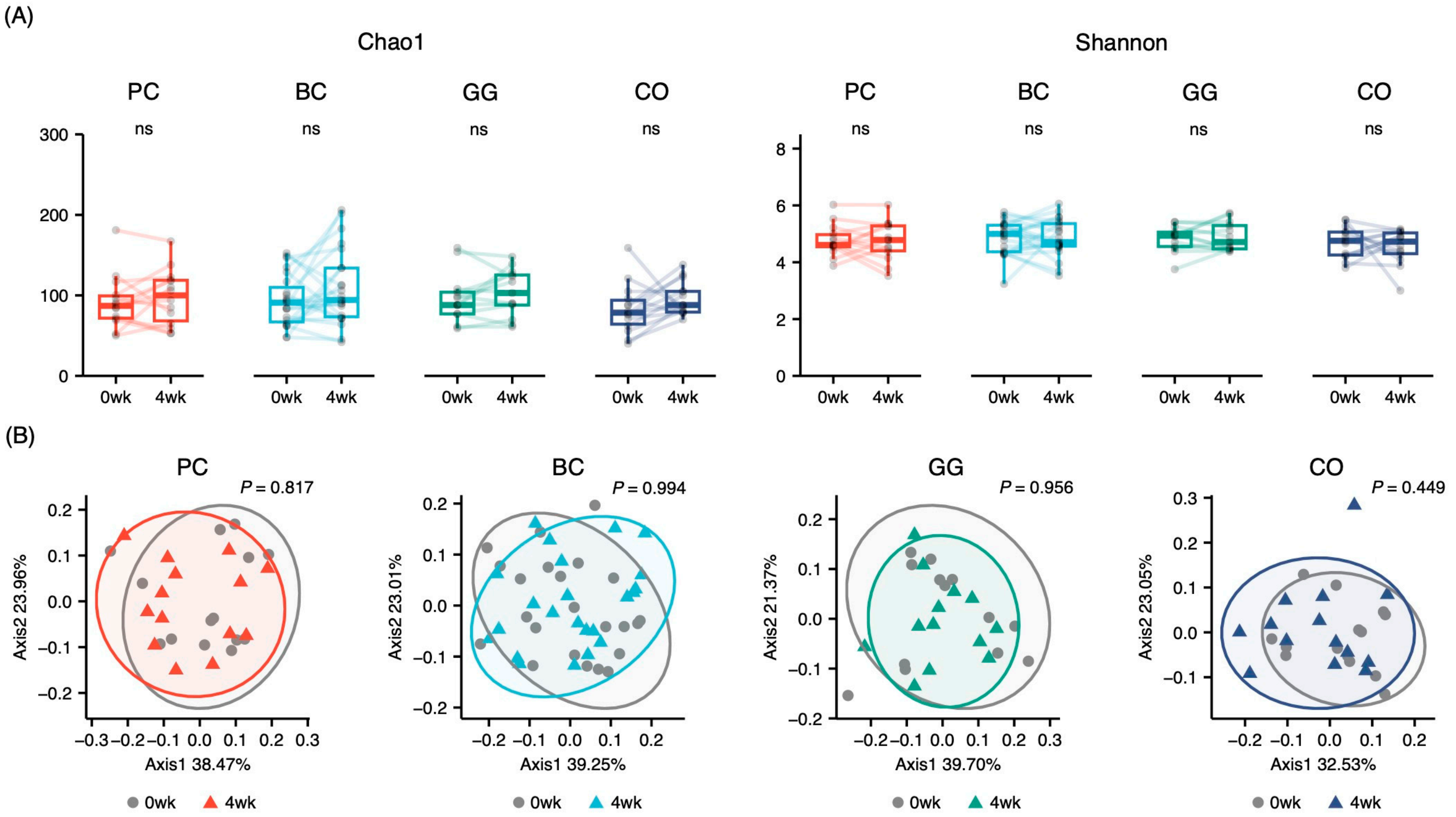

Figure S3. Subgroup analysis of the effect of blackcurrant extract and PHGG intake on the alpha- and beta- diversities of the fecal microbiotas, focusing on participants with possible gut dysbiosis. (A) Chao1 and Shannon indices of the fecal microbiotas of the respective

groups were compared between weeks 0 and 4 by a paired *t*-test. Dots connected by lines mean individual participants. (B) PCoA plots of fecal microbiotas based on weighted UniFrac distances. Gray and colored dots denote individual participants at weeks 0 and 4, respectively. Ellipses enclosing clusters mean 90% confidence interval. The differences between weeks 0 and 4 were evaluated by PERMANOVA (permutation = 9999).
